# Supplementary material for: Yiai Fuzheng decoction inhibits triple-negative breast cancer by remodeling the immune microenvironment
Source: Front Immunol. 2025 Sep 30;16:1615631. doi: 10.3389/fimmu.2025.1615631 (PMC12518410; doi:10.3389/fimmu.2025.1615631)
Supplement: Supplementary file 2 [file DataSheet1.zip › Supplementary File 2/Treatment/C_vs_D/07_Pathway_Analysis/Pathway_Analysis/Sig_Pathways_Name/Tryptophan metabolism.pdf]

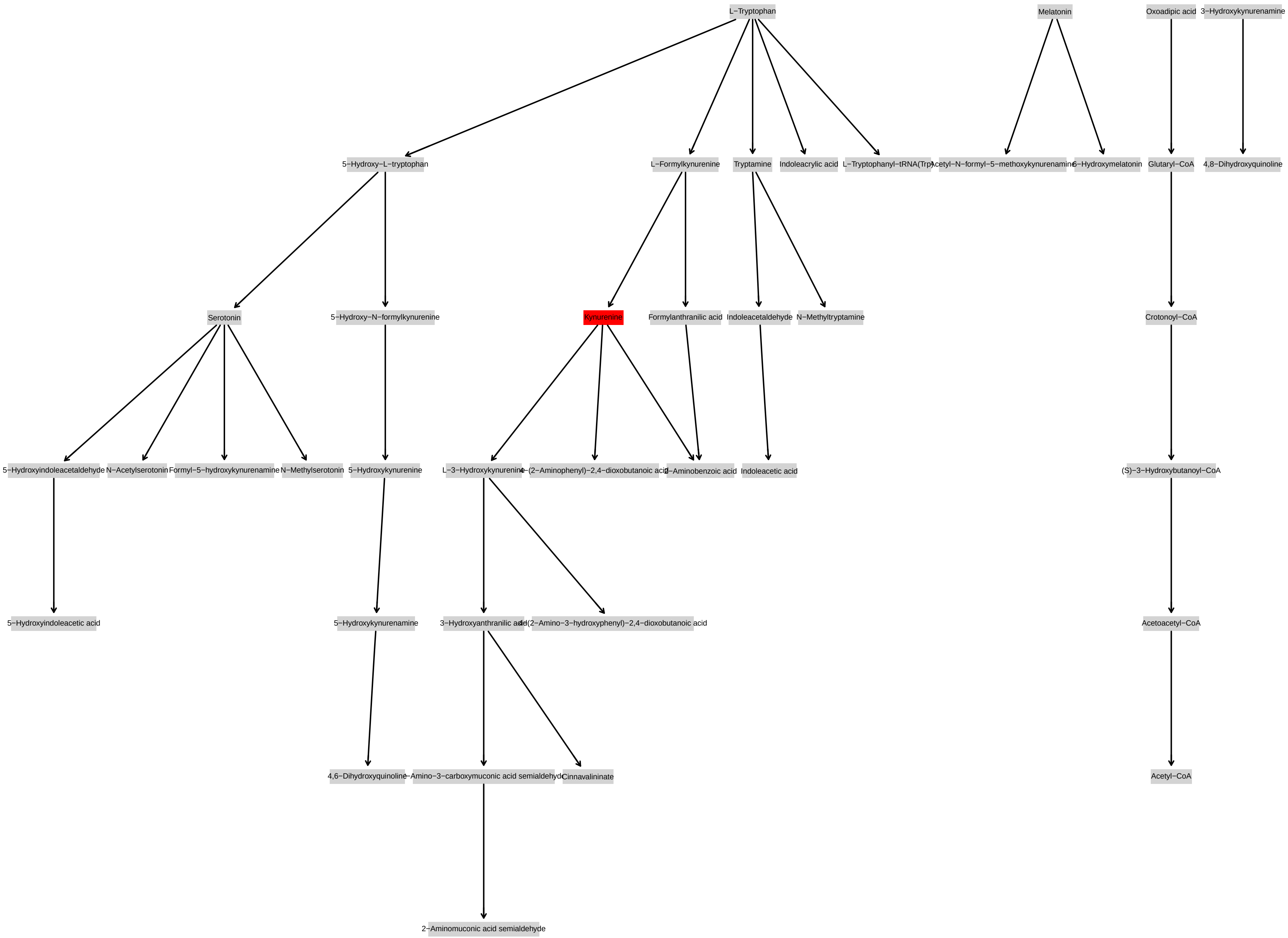
L−Tryptophan
Melatonin
Oxoadipic acid
3−Hydroxykynurenamine
5−Hydroxy−L−tryptophan
L−Formylkynurenine
Tryptamine
Indoleacrylic acid
L−Tryptophanyl−tRNA(Trp)
Acetyl−N−formyl−5−methoxykynurenamine
6−Hydroxymelatonin
Glutaryl−CoA
4,8−Dihydroxyquinoline
Serotonin
5−Hydroxy−N−formylkynurenine
Kynurenine
Formylanthranilic acid
Indoleacetaldehyde
N−Methyltryptamine
Crotonoyl−CoA
5−Hydroxyindoleacetaldehyde
N−Acetylserotonin
Formyl−5−hydroxykynurenamine
N−Methylserotonin
5−Hydroxykynurenine
L−3−Hydroxykynurenine
4−(2−Aminophenyl)−2,4−dioxobutanoic acid
2−Aminobenzoic acid
Indoleacetic acid
(S)−3−Hydroxybutanoyl−CoA
5−Hydroxyindoleacetic acid
5−Hydroxykynurenamine
3−Hydroxyanthranilic acid
4−(2−Amino−3−hydroxyphenyl)−2,4−dioxobutanoic acid
Acetoacetyl−CoA
4,6−Dihydroxyquinoline
2−Amino−3−carboxymuconic acid semialdehyde
Cinnavalininate
Acetyl−CoA
2−Aminomuconic acid semialdehyde
